# Rib myelolipoma: a case report

**DOI:** 10.1186/s13256-024-04682-1

**Published:** 2024-08-12

**Authors:** André Amate Neto, Felipe Ramos Camargo Preto, Amanda Tollini de Moraes, Sarah Lopes Salomão, Thiago Barreto Frederigue, Mariane Nunes de Nadai, Marcel Koenigkam Santos, Tales Rubens de Nadai

**Affiliations:** 1 University of São Paulo, Bauru Medical School (FMBRU-USP), Alameda Doutor Octavio Pinheiro Brisolla, 9-75, Bauru, São Paulo 17012-901 Brazil; 2 Bauru, São Paulo 17012-200 Brazil

**Keywords:** Myelolipoma, Thoracotomy, Ribs, Case report

## Abstract

**Background:**

Myelolipoma is an uncommon benign tumor composed of mature adipose tissue and hematopoietic elements. These tumors generally affect the adrenal glands, with anomalous presentations being rare and with few cases described in the literature. Most myelolipomas are asymptomatic and discovered incidentally, either through imaging tests or at autopsies. However, depending on the location and size of the lesion, myelolipomas can cause symptoms of mass effect. This article aims to report a very rare presentation of a symptomatic primary myelolipoma affecting the ribs.

**Case presentation:**

A 21-year-old white female patient presented with a complaint of burning chest pain over 3 months, with gradual worsening in intensity, accompanied by a progressively growing bulge in the right thoracic wall. The patient underwent thoracotomy of the fifth and sixth ribs with complete excision of the lesion with a safety margin. Thoracic wall reconstruction was performed using a polypropylene mesh. The patient had a good postoperative course and was discharged on postoperative day 3. Histopathological examination revealed a histological image consistent with myelolipoma.

**Conclusions:**

This report underscores the importance of considering a myelolipoma diagnosis for tumor masses in the ribs.

**Supplementary Information:**

The online version contains supplementary material available at 10.1186/s13256-024-04682-1.

## Background

Myelolipomas are rare benign tumors composed of mature adipose tissue and hematopoietic tissue that generally affect the adrenal glands. This type of lesion was first described by Gierke in 1905 and termed myelolipoma by Oberling in 1929. The number of reported cases of these tumors has increased due to enhanced diagnostic imaging techniques. The diagnosis is usually incidental, as the clinical presentation of these lesions is mostly asymptomatic, without hormonal activity. The incidence of myelolipomas at autopsies is estimated to be between 0.003% and 0.4%, with presentations outside the adrenal glands being even rarer [1–3].

Extra-adrenal involvement has few reports in the literature, with it being particularly uncommon for the ribs to be effected, with only two descriptions found in the current literature [4, 5]. In general, extra-adrenal myelolipoma consists of a well-circumscribed, slow-growing mass, generating non-specific symptoms, such as increased local volume, continuous pain, and hemorrhages [3]. These tumors are most commonly diagnosed in women aged between the fifth and seventh decade, with a female-to-male ratio of 2:1 [5].

Currently, there is still no consensus on the etiology and pathogenesis of extra-adrenal myelolipomas (EAMLPs). The absence of clinical symptoms and specific imaging features makes preoperative diagnosis challenging. Pathological examination is essential to obtain a definitive diagnosis. Due to the rarity of these tumors and the lack of reported cases, standard treatment protocols and long-term outcomes for EAMLPs remain uncertain [2, 5].

In this work, we aim to present a symptomatic case of primary myelolipoma involving the ribs.

## Case presentation

A 21-year-old white female patient presented with a complaint of burning chest pain over 3 months, gradually worsening in intensity. She also noticed a progressively growing bulge in the right thoracic wall. The patient had no underlying medical conditions and denied a history of recent illness. On physical examination, a palpable and painful mass was found at the level of the fifth and sixth ribs of the right hemithorax. Given the findings, the patient underwent a chest X-ray and, subsequently, a chest computed tomography.

**Fig. 1:**
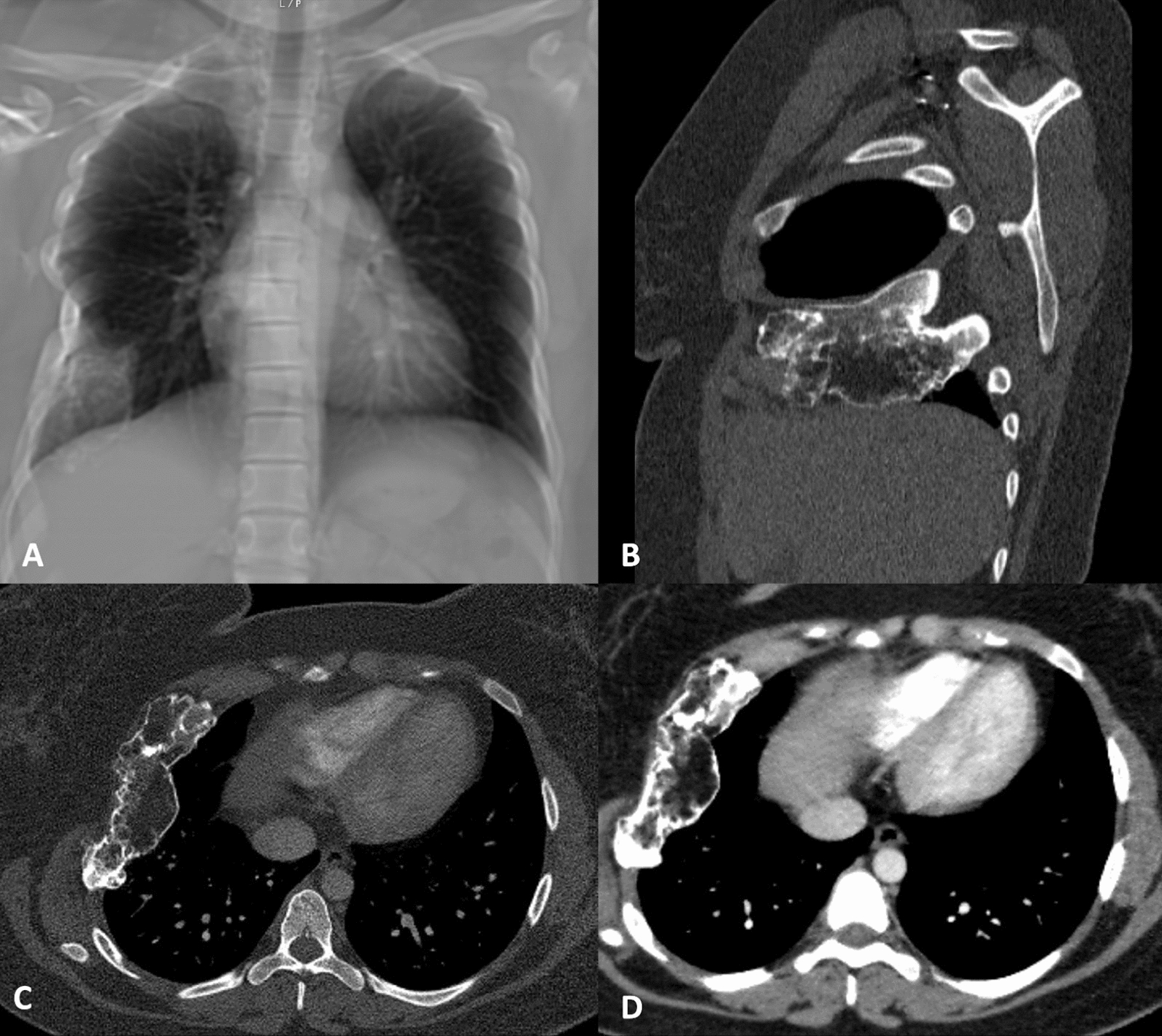
Chest X-ray (**A**) showing significant deformity of the rib cage on the right. Sagittal (**B**) and axial (**C** and **D**) sections of chest computed tomography demonstrating a lesion involving the fifth and sixth ribs, with an inflatable, lytic appearance with dense areas, including an aspect of ground glass, without an evident component of cortical rupture or associated soft tissue mass.; lesion measured 10.4 × 3.7 cm

The imaging exams revealed an important deformity of the rib cage on the right, with a 10.4 × 3.7 cm lesion with an inflatable and lytic appearance involving the fifth and sixth ribs, mainly this one. Dense areas including a ground-glass appearance were observed, with no evident component of cortical rupture or associated soft tissue mass (Fig. 1).

Due to pain that severely limited the patient’s daily activities, specialists opted for surgical removal of the tumor, without the need for prior fine-needle aspiration cytology (FNAC). The patient underwent thoracotomy of the fifth and sixth ribs with complete excision of the lesion with a safety margin. The reconstruction of the thoracic wall was performed using a polypropylene mesh.

She had a good postoperative course, showing good recovery of the surgical wound and good pain control. The patient was discharged from the hospital on the third day after surgery, with a 12-month follow-up and no recurrences or complications.

The anatomopathological result based on the resection product of the rib segments indicated a histological picture compatible with myelolipoma, without invasion of the ribs by the lesion.

**Fig. 2:**
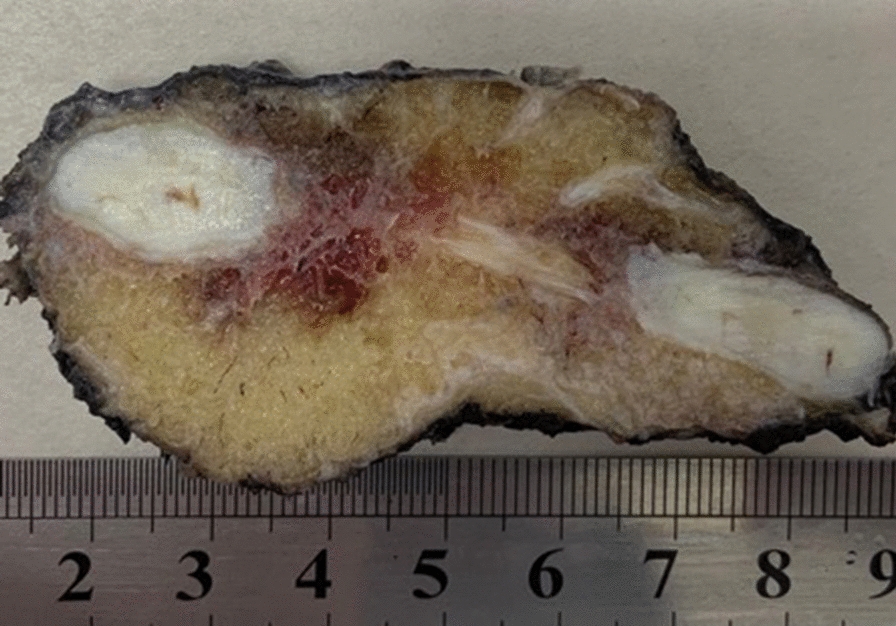
Macroscopy, sagittal section examination of the surgical specimen

Macroscopic examination of the resection product demonstrated a rib with an expansive lesion with a fatty appearance and bone lysis (Fig. 2).

**Fig. 3:**
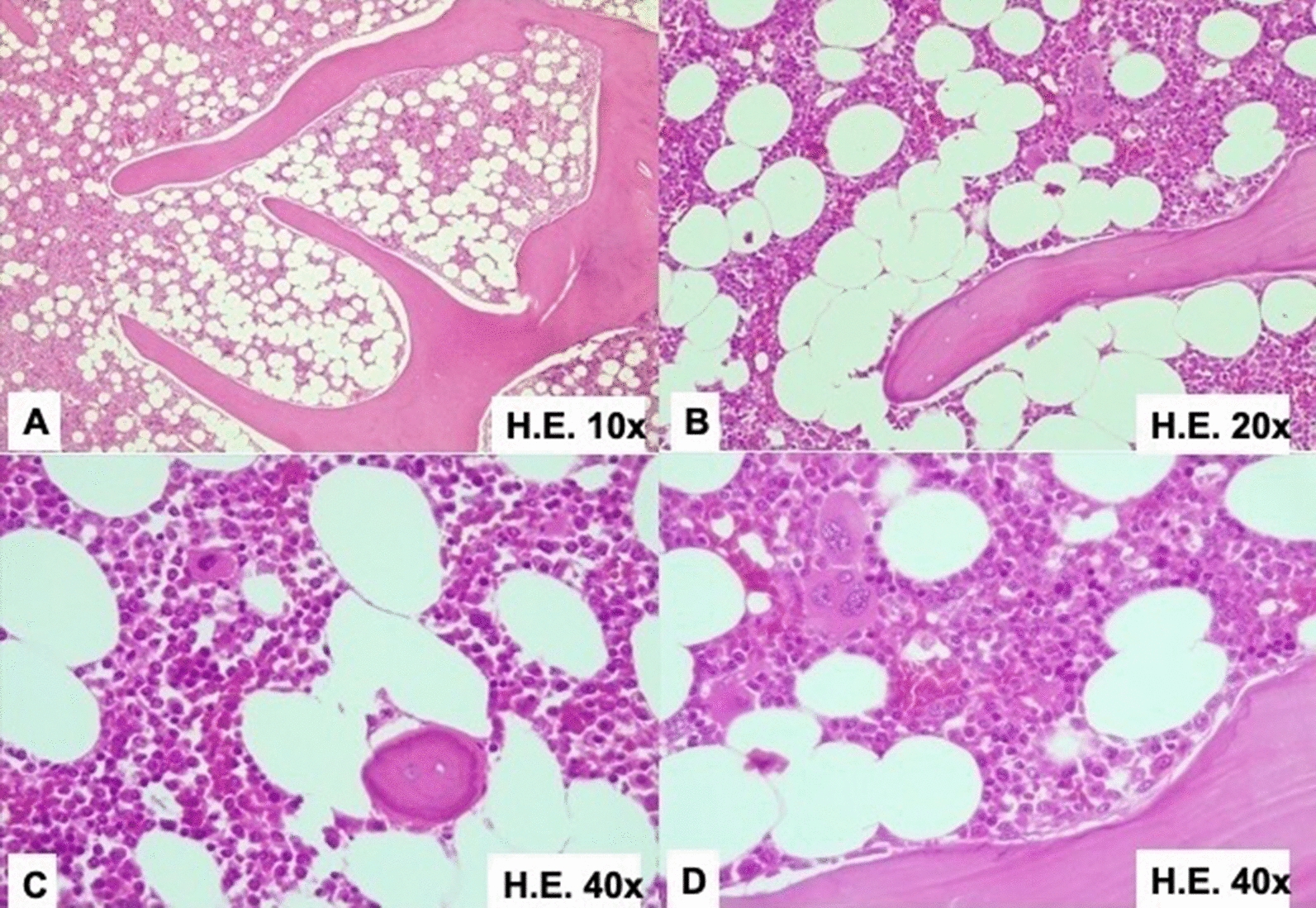
**A** 10× magnification, **B** 20× magnification, **C** 40× magnification, **D** 40× magnification (different angle); hematoxylin and eosin sections revealing a lesion composed of a proliferation of mature adipose cells together with extramedullary hematopoietic cells with complete maturation amid mature trabecular bone tissue

Upon microscopic examination of the surgical specimen, in the hematoxylin and eosin (HE) sections, a lesion composed of a proliferation of mature adipose cells (adipocytes) together with extramedullary hematopoietic cells (of the three lineages—myeloid, erotroid and megakaryocytes) with complete maturation (similar to a hypercellular bone marrow) amid trabecular mature bone tissue was observed (Fig. 3).

## Discussion and conclusions

Myelolipoma is an uncommon benign tumor, composed of mature adipose tissue and hematopoietic elements [4, 6]. In most cases, myelolipomas affect the adrenal glands, and lesions in other locations, such as kidneys, spleen, and bones, are rare, with few reports in the literature [4, 5, 7]. Extra-adrenal myelolipomas represent approximately 15% of all myelolipomas, with the presacral cavities being the most affected (40%), followed by the peritoneal (20%) and thoracic cavities (15%) [5, 8]. Affection of the ribs is especially rare, considering that, in our review of the literature, we identified only two reports of primary involvement of the ribs [4, 5].

Myelolipoma is pathologically characterized by the presence of adipocytes and cells normally found in bone marrow. The relative proportion of these two types of cells varies among tumors. Bone marrow cells typically found in these masses include megakaryocytes and immature white blood cells. Large lesions may evolve with bleeding inside them; on microscopy, red blood cells or hemosiderin can be observed within the tumor. Large open spaces called cysts may also be observed, especially in larger tumors.

Cao *et al*. [5] reported a series of 11 cases of extra-adrenal myelolipoma (EAMLPs), with 7 in the thoracic spine and 4 in intraosseous regions. Among the intraosseous cases, one of the reported cases involved the right eighth posterior rib in a 55-year-old woman who had been experiencing discomfort for 1 month. The tumor measured 4.4 cm and the surgery lasted 70 minutes, with a 37-month follow-up [5]. Meanwhile, Wen *et al*. [4] reported a case of a 2.0 × 2.0 × 1.5 cm myelolipoma adherent to the sixth ribs of an 18-year-old man, with a 24-month follow-up [4].

**Table:** 

Reference	Affected rib	Patient’s gender	Pacient’s age	Chief complaint	Myelolipomas’s size (cm)	Total follow up (months)
Wen *et al*. [4]	Sixth ribs	Man	18	Mass found in the left anterior chest during a routine physical exam. No further complaints	2.0 × 2.0 × 1.5	24
Cao *et al*. [5]	Right eighth posterior rib	Woman	55	Discomfort in the region for 1 month	4.4	37
Our case	Fifth and sixth right ribs	Woman	21	Burning chest pain for more than 3 months, gradually worsening in intensity	10.4 × 3.7	12

The etiopathogenesis of myelolipomas is not well defined. These tumors have been related to increased concentrations of adrenocorticotropic hormone (e.g., Cushing’s syndrome, Addison’s syndrome, adrenal adenoma, 21-hydroxylase deficiency) or to conditions of chronic stress caused by infection, necrosis, or inflammation [5, 9]. A frequent association with obesity, type 2 diabetes mellitus, and hypertension has been observed [9–11]. Some explanations suggest that extra-adrenal myelolipomas may result from metaplasia of embryonic cells, embolization of bone marrow tissue, or metaplasia of adrenal cortical cells. [5, 9, 12, 13].

In most cases, the diagnosis of EAMLPs is incidental, discovered in imaging studies or autopsies, as patients are usually asymptomatic [9, 13, 14]. However, depending on the location and size of the tumor, some patients may experience pain due to mechanical compression, as in the reported case, or even bleeding or tumor infarction [5, 9, 14].

Although computed tomography and magnetic resonance imaging contribute to the detection of extra-adrenal myelolipomas, establishing a definitive diagnosis for rare EAMLPs on the sole basis of imaging is difficult [15]. Histopathological study is essential for obtaining a definitive diagnosis [13].

EAMLPs generally present well-defined masses with an approximate diameter of 5.9 cm (range 1.5–25 cm) [15, 16]. On computed tomography, the mass has a low attenuation value consistent with macroscopic adipose tissue (less than −20 HU), but may have soft tissue density elements representing myeloid tissue [15, 17]. Administration of intravenous contrast agent may enhance hematopoietic soft-tissue components [14]. Calcifications and hemorrhages may also be observed [1]. Although these tumors can be large, there is no invasion into surrounding structures [14]. Nevertheless, depending on the size of the lesion, there may be displacement of adjacent structures [14]. On T1- and T2-weighted magnetic resonance imaging, EAMLPs show high signal intensity for fatty elements. The signal of hematopoietic components is low on T1-weighted images and moderate on T2-weighted images [15]. Soft tissue elements can be enhanced by injecting gadolinium-based contrast agents [14, 16].

Differential diagnoses of EAMLPs include lipoma, liposarcoma, and extramedullary hematopoiesis [13, 18]. Lipoma is a benign tumor, composed predominantly of mature adipose tissue, which can be differentiated from myelolipoma on the basis of extensive sampling [18]. Liposarcoma is a fatty tissue tumor that, despite presenting radiological characteristics similar to myelolipoma, does not present bone marrow elements and has a manifestation of infiltrative growth, being composed of large and atypical cells mixed with lipoblasts and adipocytes [18, 19]. Extramedullary hematopoiesis is generally associated with the presence of myeloproliferative disorders or compensatory manifestations of various types of chronic anemia [13, 18]. It is mainly made up of hematopoietic cells and erythroid hyperplasia without fatty tissue elements [18]. Unlike the presentation of EAMPLs, in extramedullary hematopoiesis there is usually the presence of chronic anemia or hepatosplenomegaly [13, 18].

The treatment of extra-adrenal myelolipomas is generally observational or surgical [5, 9]. In most cases, surgery is recommended in symptomatic presentations or in the presence of larger masses (> 7 cm), with mechanical compression of adjacent structures or risk of rupture and hemorrhage [5, 9, 12]. Surgical procedures may also be indicated to obtain a definitive diagnosis of the anomaly and to exclude malignancy [9]. Depending on the size and location of each lesion, tumors affecting thoracic cavities can be resected by video-assisted thoracoscopic surgery (VATS) or by thoracotomy [15], as occurred in our case. If the lesion is small (< 4 cm) and the patient is asymptomatic, the tumor can be conservatively managed [9, 12].

Myelolipoma is a rare benign tumor with no defined etiology that most commonly affects the adrenal glands. The reported case of rib involvement is an especially rare presentation, and is the third case described in the literature to date. Finally, this case demonstrates the importance of considering a myelolipoma diagnosis for tumor masses in the ribs.

### Supplementary Information

Additional file 1. CARE Checklist. Application of the CARE Checklist in this case report.

## Data Availability

All data generated or analyzed during this study are included in this published article [and its supplementary information files].

## Abbreviations

FNAC: Fine-needle aspiration cytology
HE: Hematoxylin and eosin
EAMLPs: Extra-adrenal myelolipomas
VATS: Video-assisted thoracoscopic surgery
