# Supplementary material for: Rib myelolipoma: a case report
Source: J Med Case Rep. 2024 Aug 12;18:377. doi: 10.1186/s13256-024-04682-1 (PMC11318312; doi:10.1186/s13256-024-04682-1)
Supplement: Supplementary file 1 — Additional file 1. CARE Checklist. Application of the CARE Checklist in this case report. [file 13256_2024_4682_MOESM1_ESM.pdf]

CARE Checklist of information to include when writing a case report

CARE case report guidelines

(cc) BY-NC-ND

Topic	Item	Checklist item description	Reported on Line
Title	1	The diagnosis or intervention of primary focus followed by the words "case report"	1
Key Words	2	2 to 5 key words that identify diagnoses or interventions in this case report, including "case report"	51
Abstract (no references)	3a	Introduction: What is unique about this case and what does it add to the scientific literature?	34-40
	3b	Main symptoms and/or important clinical findings	41-43
	3c	The main diagnoses, therapeutic interventions, and outcomes	42-46
	3d	Conclusion—What is the main "take-away" lesson(s) from this case?	48-49
Introduction	4	One or two paragraphs summarizing why this case is unique (**may include references**)	55-73
Patient Information	5a	De-identified patient specific information.	77
	5b	Primary concerns and symptoms of the patient.	77-79
	5c	Medical, family, and psycho-social history including relevant genetic information	79-80
	5d	Relevant past interventions with outcomes	Not applicable
Clinical Findings	6	Describe significant physical examination (PE) and important clinical findings.	80-81
Timeline	7	Historical and current information from this episode of care organized as a timeline	77-97
Diagnostic Assessment	8a	Diagnostic testing (such as PE, laboratory testing, imaging, surveys).	83-86, 98-109
	8b	Diagnostic challenges (such as access to testing, financial, or cultural)	Not applicable
	8c	Diagnosis (including other diagnoses considered)	98-99
	8d	Prognosis (such as staging in oncology) where applicable	Not applicable
Therapeutic Intervention	9a	Types of therapeutic intervention (such as pharmacologic, surgical, preventive, self-care)	90-94
	9b	Administration of therapeutic intervention (such as dosage, strength, duration)	90-94
	9c	Changes in therapeutic intervention (with rationale)	Not applicable
Follow-up and Outcomes	10a	Clinician and patient-assessed outcomes (if available)	95-97
	10b	Important follow-up diagnostic and other test results	95-97
	10c	Intervention adherence and tolerability (How was this assessed?)	95-97
	10d	Adverse and unanticipated events	Not applicable
Discussion	11a	A scientific discussion of the strengths AND limitations associated with this case report	115-122
	11b	Discussion of the relevant medical literature **with references**.	115-183
	11c	The scientific rationale for any conclusions (including assessment of possible causes)	115-183
	11d	The primary "take-away" lessons of this case report (without references) in a one paragraph conclusion	184-187
Patient Perspective	12	The patient should share their perspective in one to two paragraphs on the treatment(s) they received	95-97
Informed Consent	13	Did the patient give informed consent? Please provide if requested	Yes ☒ No ☐
